# Supplementary material for: Learning from Heterogeneous Data Sources: An Application in Spatial Proteomics
Source: PLoS Comput Biol. 2016 May 13;12(5):e1004920. doi: 10.1371/journal.pcbi.1004920 (PMC4866734; doi:10.1371/journal.pcbi.1004920)
Supplement: S1 File — Visualisations of the k-NN transfer learning results for human, plant callus, plant roots and fly datasets. Including bubble plots displaying the distribution of the optimised class weights, principal components analysis plots of the LOPIT primary datasets boxplots displaying the estimated generalisation performance of each classifier. (PDF) [file pcbi.1004920.s001.pdf]

# S1 File: Results - $k$-NN Transfer Learning

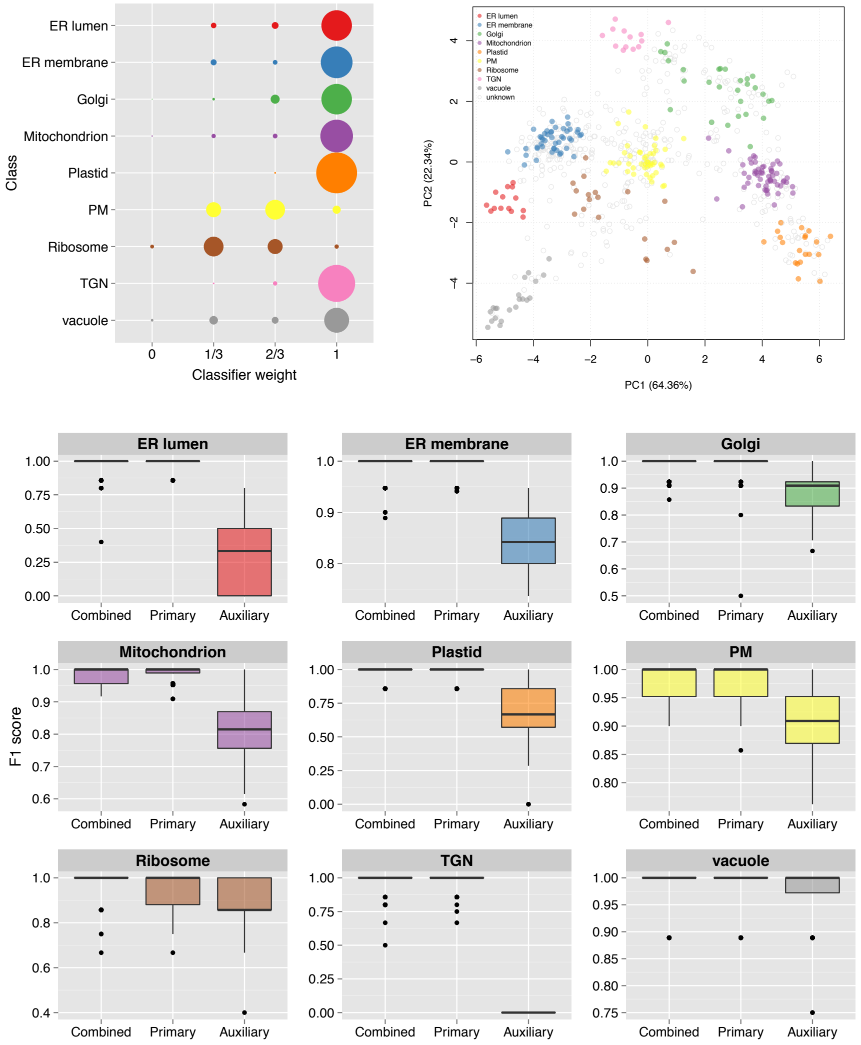


**S1 File. Fig. A. $k$-NN transfer learning results for the plant callus dataset** Top left: Bubble plot, displaying the distribution of the optimised class weights over the 100 test partitions for the transfer learning algorithm applied to the plant callus LOPIT and GO CC datasets. Top right: Principal components analysis plot of the LOPIT data (first and second components, of the possible sixteen), showing the clustering of proteins according to their density gradient distributions. Bottom: Sub-cellular class-specific box plots, displaying the estimated generalisation performance over 100 test partitions for the transfer learning algorithm applied with (i) optimised class-specific weights (combined, both LOPIT and GO CC), (ii) only primary LOPIT data and (iii) only auxiliary GO CC data, for each sub-cellular class.

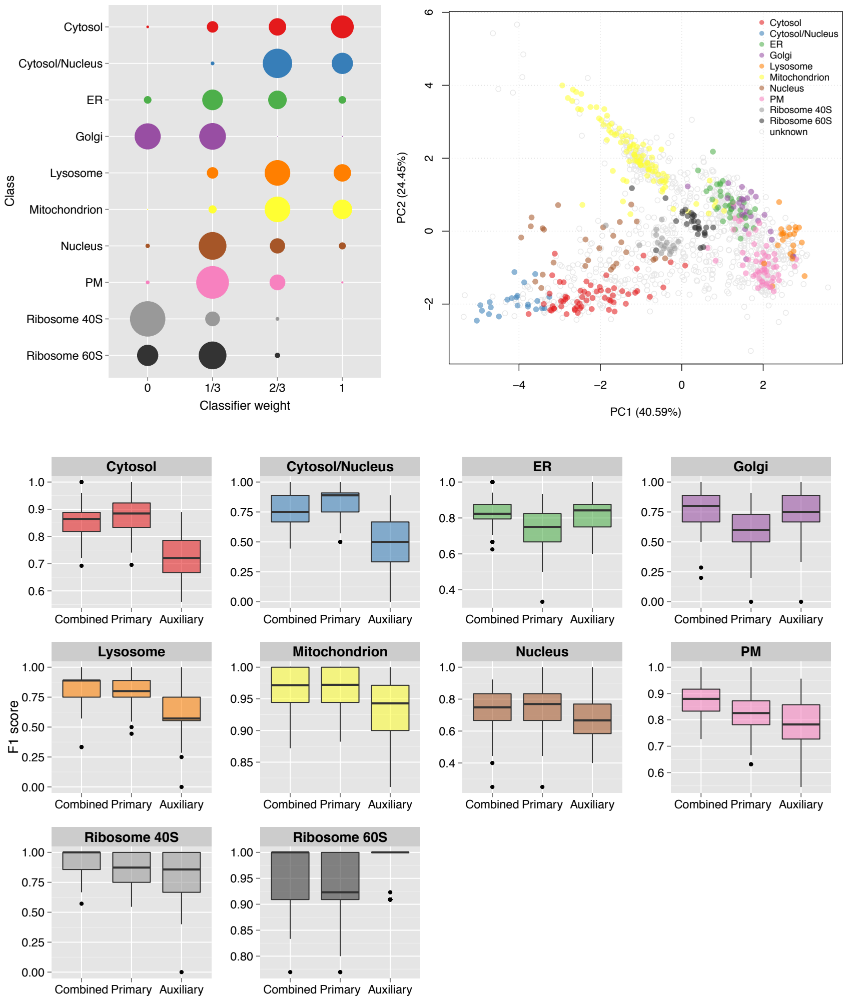


**S1 File. Fig. B.** ***k*-NN transfer learning results for the human dataset** Top left: Bubble plot, displaying the distribution of the optimised class weights over the 100 test partitions for the transfer learning algorithm applied to the plant callus LOPIT and GO CC datasets. Top right: Principal components analysis plot of the LOPIT data (first and second components, of the possible eight), showing the clustering of proteins according to their density gradient distributions. Bottom: Sub-cellular class-specific box plots, displaying the estimated generalisation performance over 100 test partitions for the transfer learning algorithm applied with (i) optimised class-specific weights (combined, both LOPIT and GO CC), (ii) only primary LOPIT data and (iii) only auxiliary GO CC data, for each sub-cellular class.

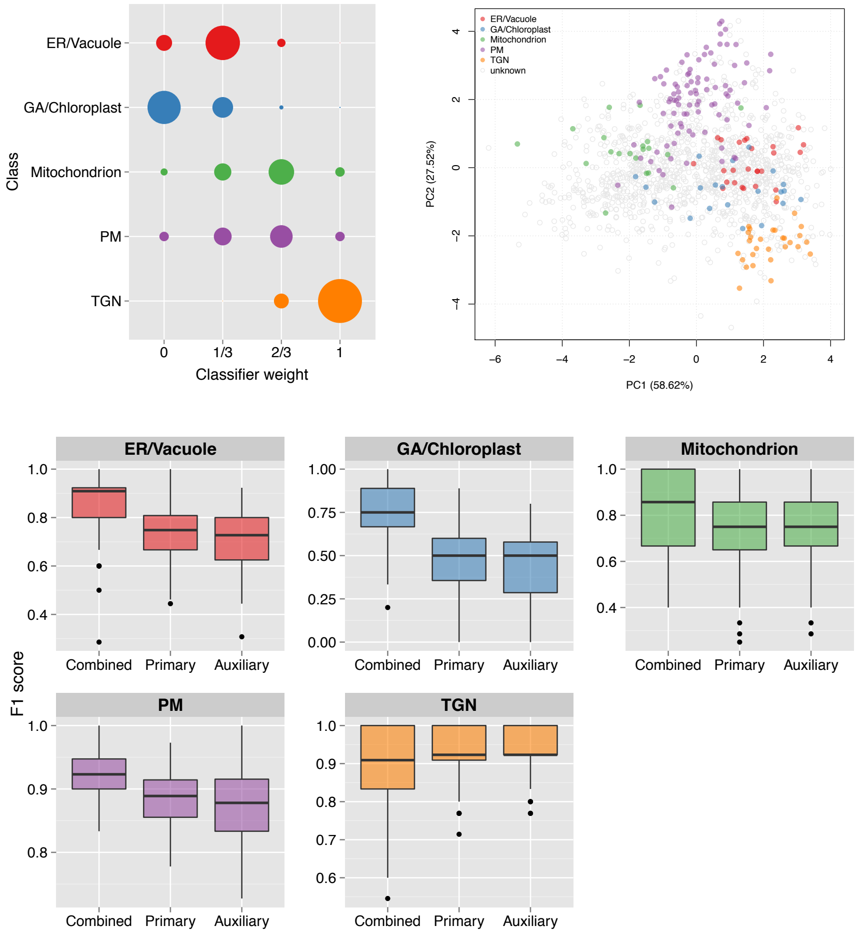


**S1 File. Fig. C. $k$-NN transfer learning results for the plant roots dataset** Top left: Bubble plot, displaying the distribution of the optimised class weights over the 100 test partitions for the transfer learning algorithm applied to the plant callus LOPIT and GO CC datasets. Top right: Principal components analysis plot of the LOPIT data (first and second components, of the possible six), showing the clustering of proteins according to their density gradient distributions. Bottom: Sub-cellular class-specific box plots, displaying the estimated generalisation performance over 100 test partitions for the transfer learning algorithm applied with (i) optimised class-specific weights (combined, both LOPIT and GO CC), (ii) only primary LOPIT data and (iii) only auxiliary GO CC data, for each sub-cellular class.

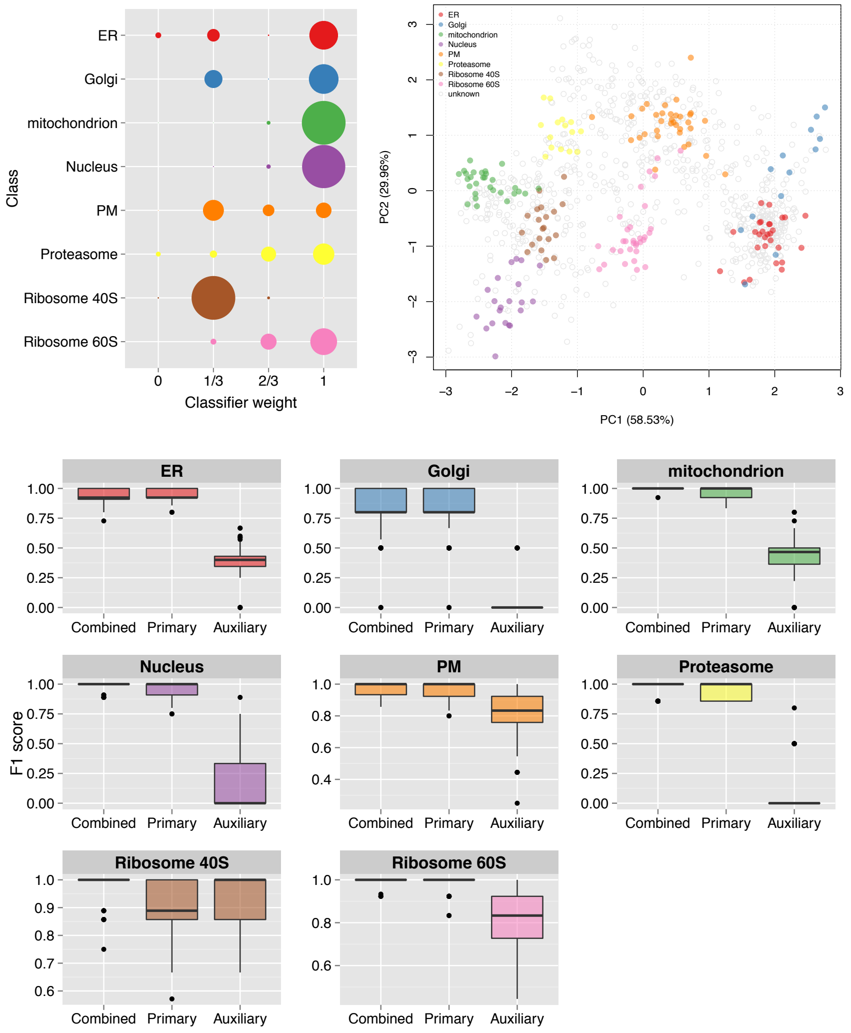


**S1 File. Fig. D. $k$-NN transfer learning results for the fly dataset** Top left: Bubble plot, displaying the distribution of the optimised class weights over the 100 test partitions for the transfer learning algorithm applied to the plant callus LOPIT and GO CC datasets. Top right: Principal components analysis plot of the LOPIT data (first and second components, of the possible four), showing the clustering of proteins according to their density gradient distributions. Bottom: Sub-cellular class-specific box plots, displaying the estimated generalisation performance over 100 test partitions for the transfer learning algorithm applied with (i) optimised class-specific weights (combined, both LOPIT and GO CC), (ii) only primary LOPIT data and (iii) only auxiliary GO CC data, for each sub-cellular class.
