# Supplementary material for: Learning from Heterogeneous Data Sources: An Application in Spatial Proteomics
Source: PLoS Comput Biol. 2016 May 13;12(5):e1004920. doi: 10.1371/journal.pcbi.1004920 (PMC4866734; doi:10.1371/journal.pcbi.1004920)
Supplement: S2 File — Boxplots displaying the estimated generalisation performance of the SVM transfer learning algorithm applied to the mouse, human, plant callus, plant roots and fly datasets. (PDF) [file pcbi.1004920.s002.pdf]

S2 File: Results - SVM Transfer Learning

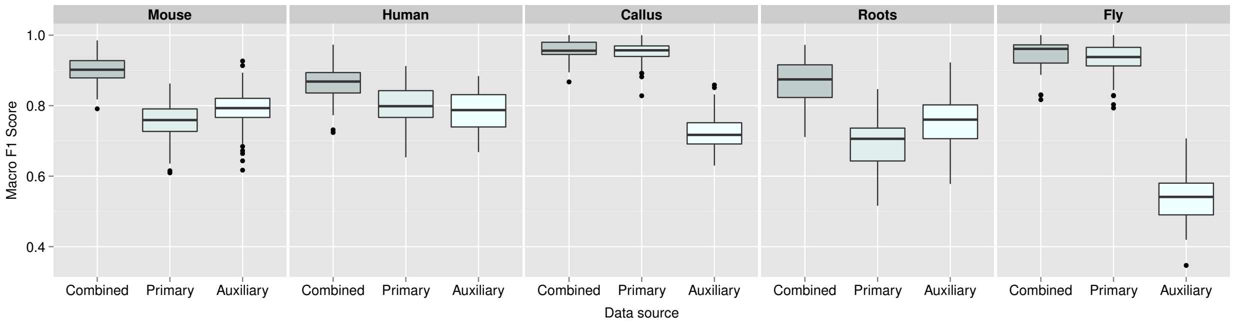

S2 File. Fig. A. Boxplots, displaying the estimated generalisation performance over 100 test partitions. Results for the SVM TL algorithm applied with (i) both primary and auxiliary data sources (combined), (ii) primary data only, and (iii) auxiliary data only, for each dataset.

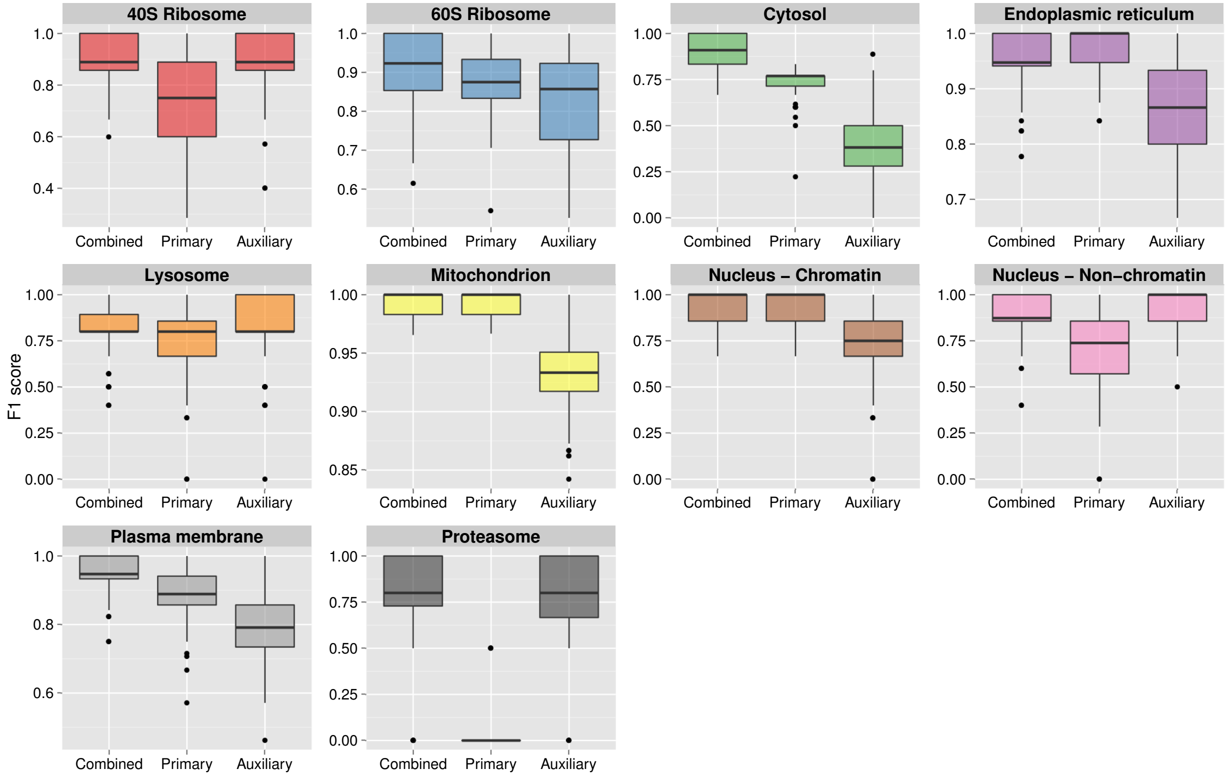

S2 File. Fig. B. SVM transfer learning results on the mouse dataset. Sub-cellular class-specific box plots, displaying the estimated generalisation performance over 100 test partitions for the SVM transfer learning algorithm applied to the mouse LOPIT and GO CC datasets with (i) optimised class-specific weights (combined), (ii) only primary data and (iii) only auxiliary data, for each sub-cellular class.

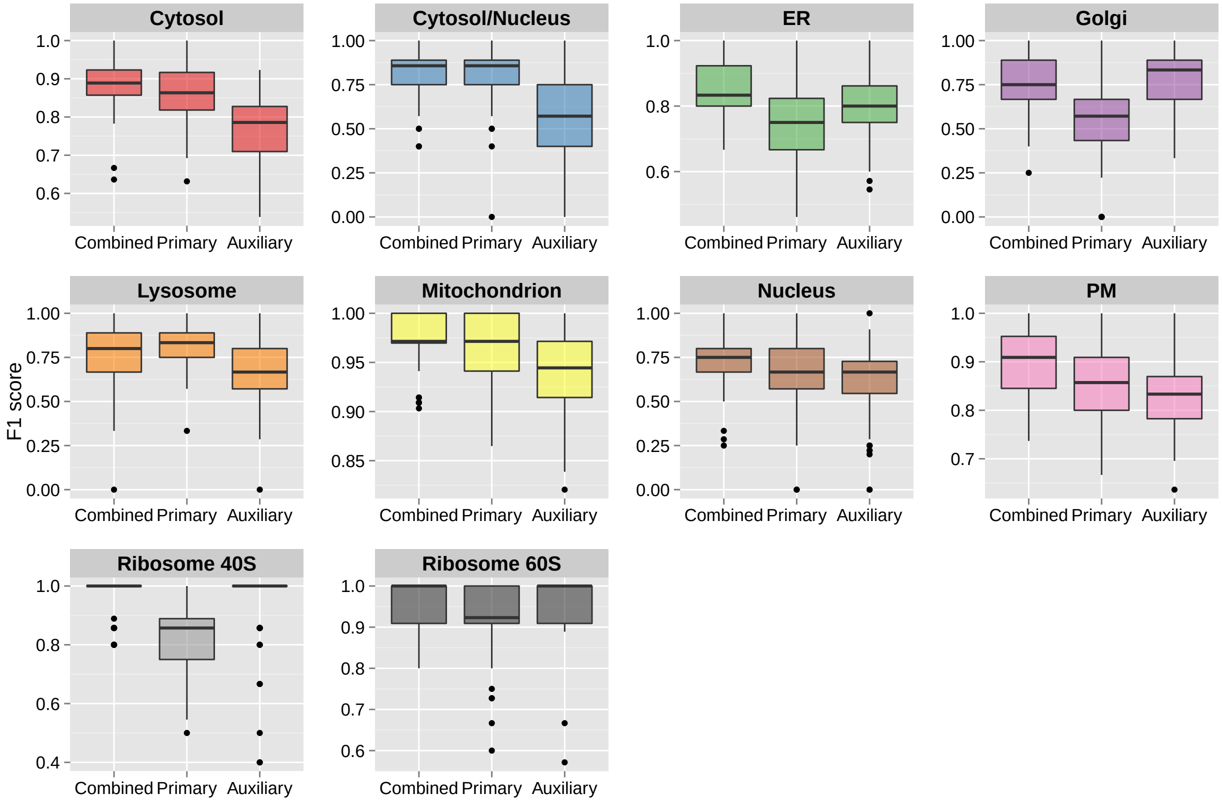

S2 File. Fig. C. SVM transfer learning results on the human dataset. Sub-cellular class-specific box plots, displaying the estimated generalisation performance over 100 test partitions for the SVM transfer learning algorithm applied to the human LOPIT and GO CC datasets with (i) optimised class-specific weights (combined), (ii) only primary data and (iii) only auxiliary data, for each sub-cellular class.

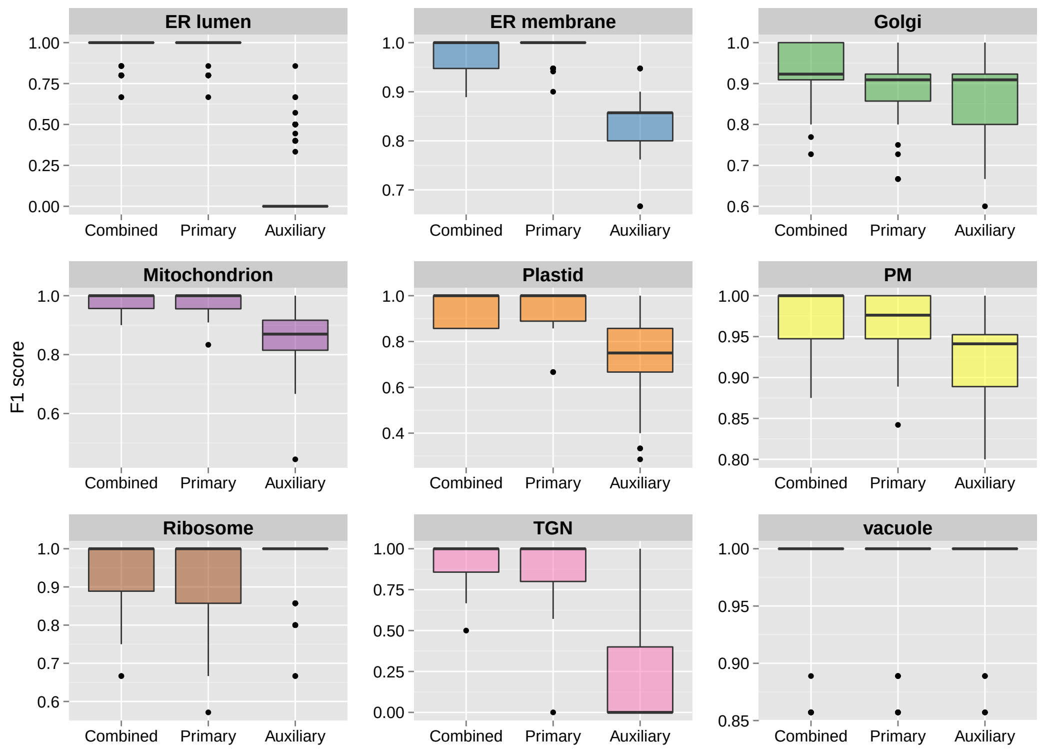

S2 File. Fig. D. SVM transfer learning results on the plant callus dataset. Boxplots show the estimated class-specific generalisation performance over 100 test partitions for the SVM transfer learning algorithm applied to the plant callus LOPIT and GO CC datasets with (i) optimised class-specific weights (combined), (ii) only primary data and (iii) only auxiliary data, for each sub-cellular class.

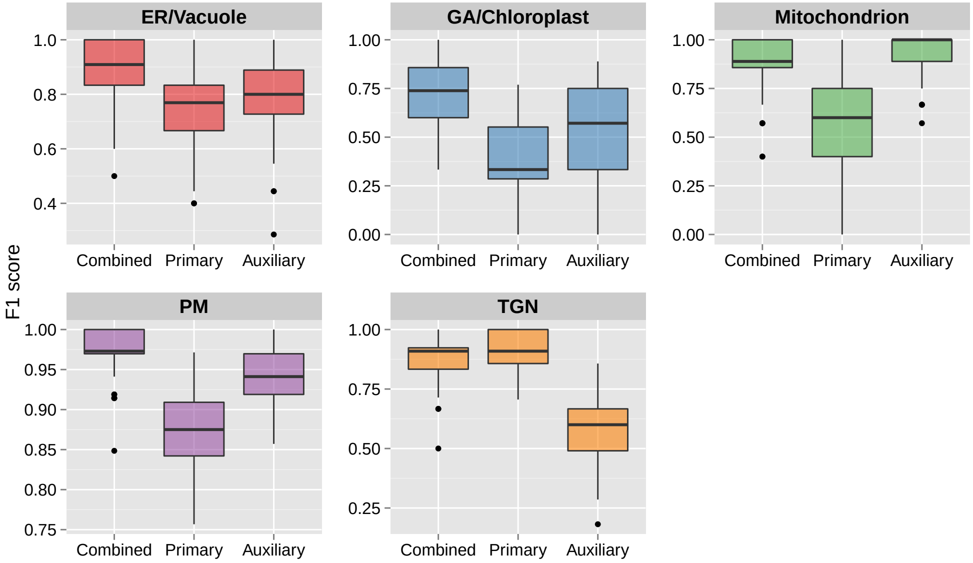

S2 File. Fig. E. SVM transfer learning results on the plant roots dataset. Sub-cellular class-specific box plots, displaying the estimated generalisation performance over 100 test partitions for the SVM transfer learning algorithm applied to the plant roots LOPIT and GO CC datasets with (i) optimised class-specific weights (combined), (ii) only primary data and (iii) only auxiliary data, for each sub-cellular class.

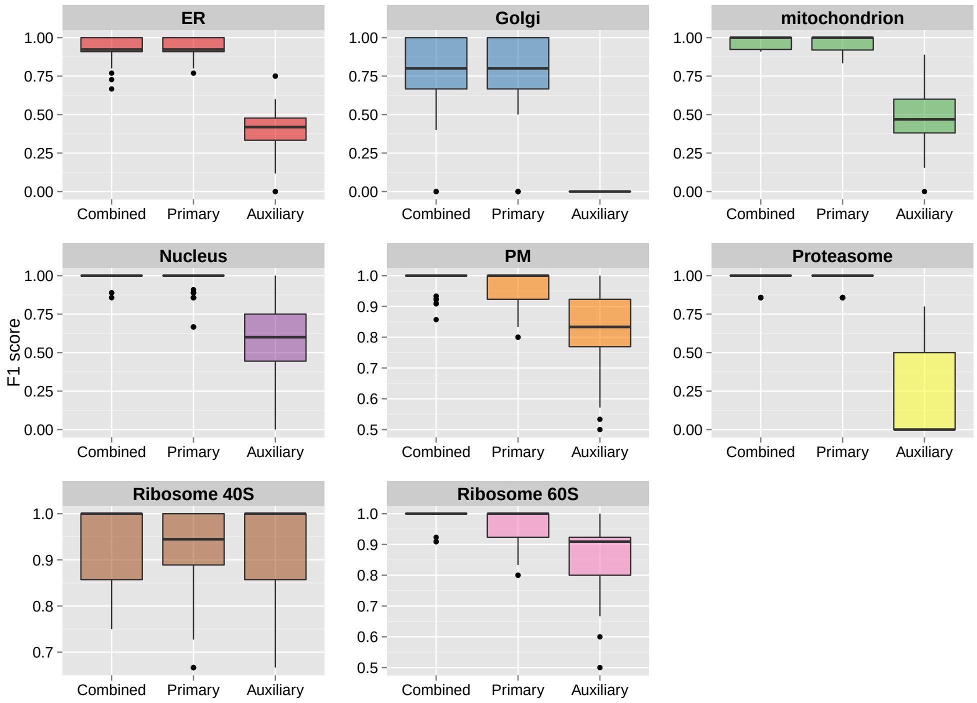

S2 File. Fig. F. SVM transfer learning results on the fly dataset. Sub-cellular class-specific box plots, displaying the estimated generalisation performance over 100 test partitions for the SVM transfer learning algorithm applied to the fly LOPIT and GO CC datasets with (i) optimised class-specific weights (combined), (ii) only primary data and (iii) only auxiliary data, for each sub-cellular class.
