# Supplementary material for: Learning from Heterogeneous Data Sources: An Application in Spatial Proteomics
Source: PLoS Comput Biol. 2016 May 13;12(5):e1004920. doi: 10.1371/journal.pcbi.1004920 (PMC4866734; doi:10.1371/journal.pcbi.1004920)
Supplement: S3 File — Macro- and class-specific results for the k-NN transfer learning algorithm used with the auxiliary Human Protein Atlas dataset, a YLoc sequence and annotation features auxiliary dataset and a protein-protein interactions dataset. (PDF) [file pcbi.1004920.s003.pdf]

S3 File: Supporting figures for other auxiliary data sources

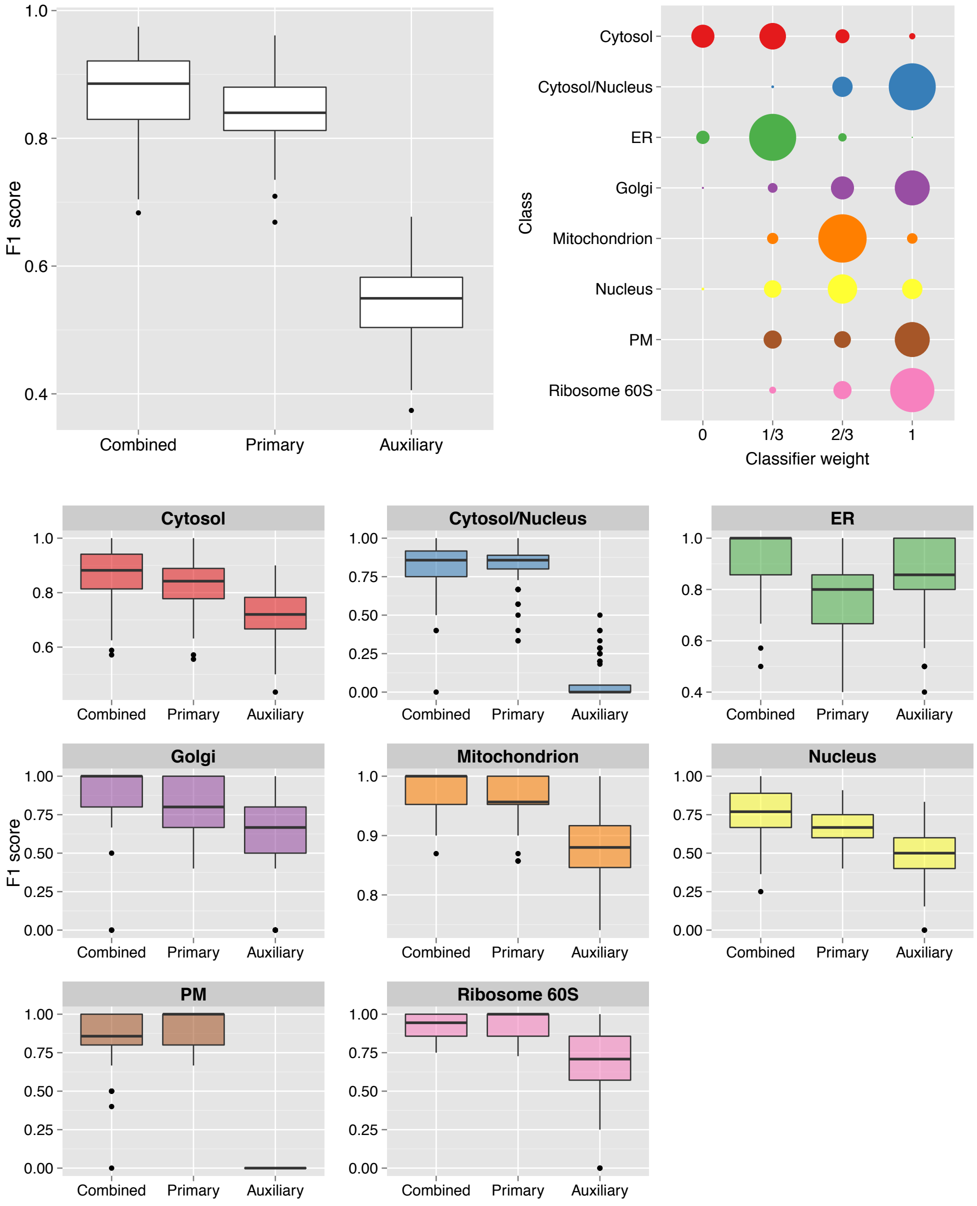

S3 File. Fig. A. The Human Protein Atlas. Top left: Boxplot displaying the macro F1 scores over the 100 test partitions for the k-NN transfer learning algorithm applied with (i) optimised class-specific weights (combined), (ii) only primary data and (iii) only auxiliary data for the human dataset. Top right: Bubble plot, displaying the distribution of the optimised class weights over the 100 test partitions for the transfer learning algorithm. Bottom: Boxplots, displaying the class specific generalisation performance over 100 test partitions for the k-NN TL experiments

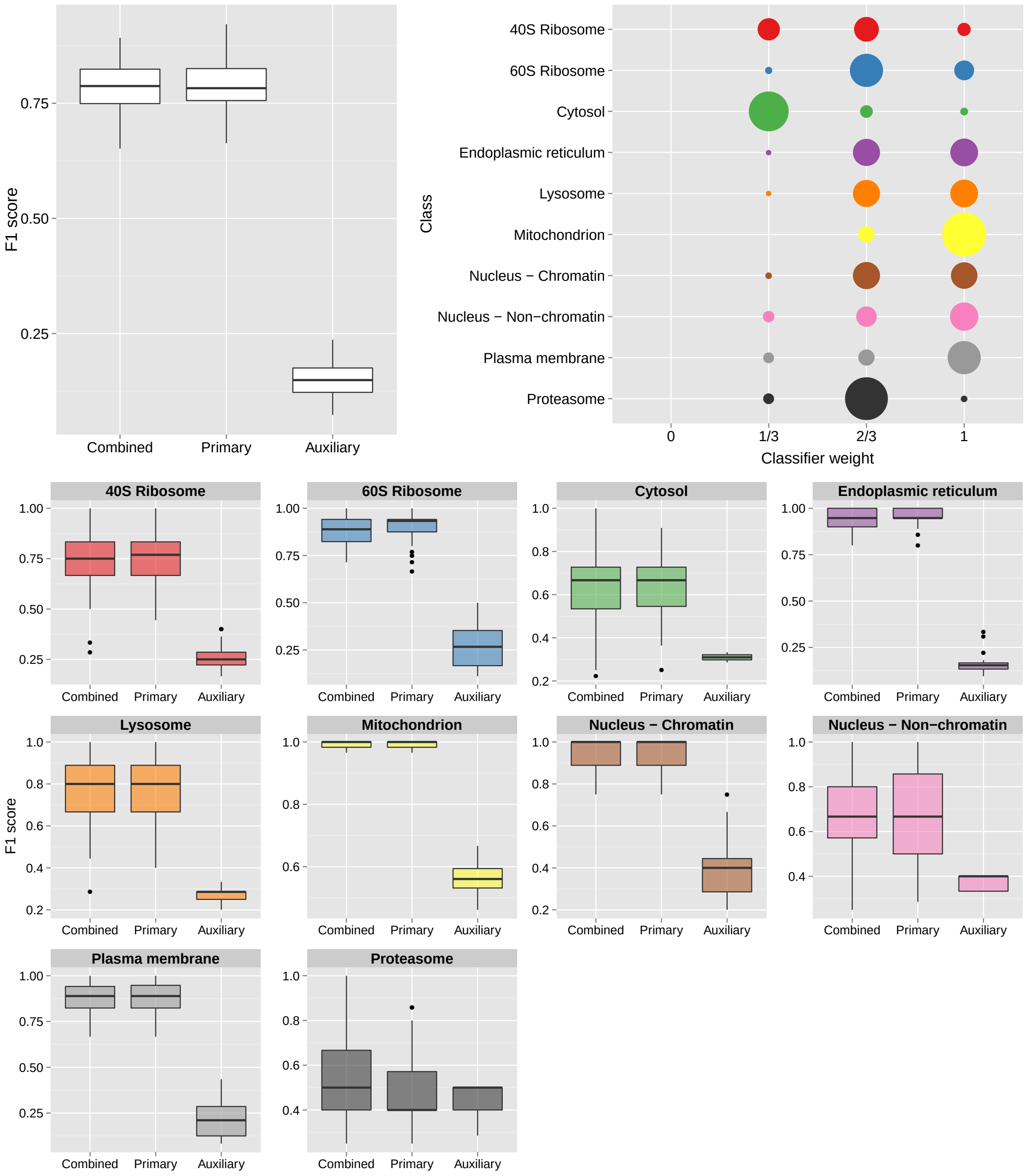

S3 File. Fig. B. YLoc Sequence and Annotation Features. Boxplots, displaying the overall (A) and class specific (C) estimated generalisation performance over 100 test partitions for the *k*-NN transfer learning (TL) algorithm applied with (i) optimised class-specific weights (combined), (ii) only primary data and (iii) only auxiliary YLoc data, for the mouse dataset. (B) Bubble plot, displaying the distribution of the optimised class weights over the 100 test partitions for the *k*-NN TL algorithm.

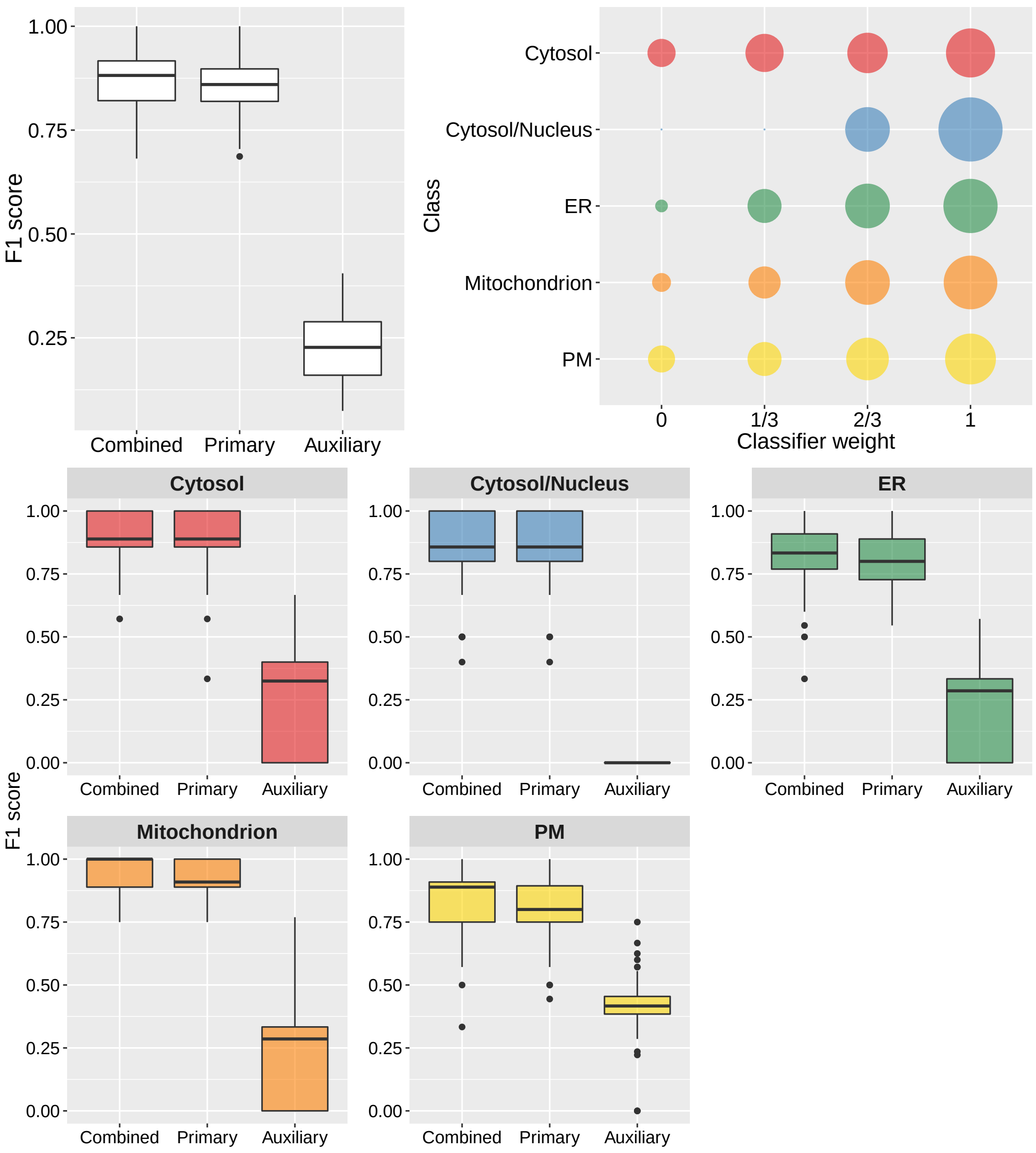

S3 File. Fig. C. Protein-protein interaction data. Boxplots, displaying the overall (A) and class specific (C) estimated generalisation performance over 100 test partitions for the k-NN transfer learning (TL) algorithm applied with (i) optimised class-specific weights (combined), (ii) only primary data and (iii) only auxiliary protein-protein interaction data, for the human dataset. (B) Bubble plot, displaying the distribution of the optimised class weights over the 100 test partitions for the k-NN TL algorithm.
