# Supplementary material for: Learning from Heterogeneous Data Sources: An Application in Spatial Proteomics
Source: PLoS Comput Biol. 2016 May 13;12(5):e1004920. doi: 10.1371/journal.pcbi.1004920 (PMC4866734; doi:10.1371/journal.pcbi.1004920)
Supplement: S4 File — Boxplots displaying the distribution of classification scores assigned to the unknown proteins in the mouse dataset for each of the 4 classifiers. Principal components analysis plot displaying the protein classification results from applying the k-NN transfer learning algorithm on the unlabelled data in the mouse dataset. Accompanying tables showing the number of sub-cellular assignments of the unlabelled proteins amongst the 10 known sub-cellular classes in the data from applying each transfer learning method. the mouse stem cell dataset highlighting the new localistions found by the k-NN transfer learning method. (PDF) [file pcbi.1004920.s004.pdf]

S4 File: Biological Application - Supporting Figures and Tables

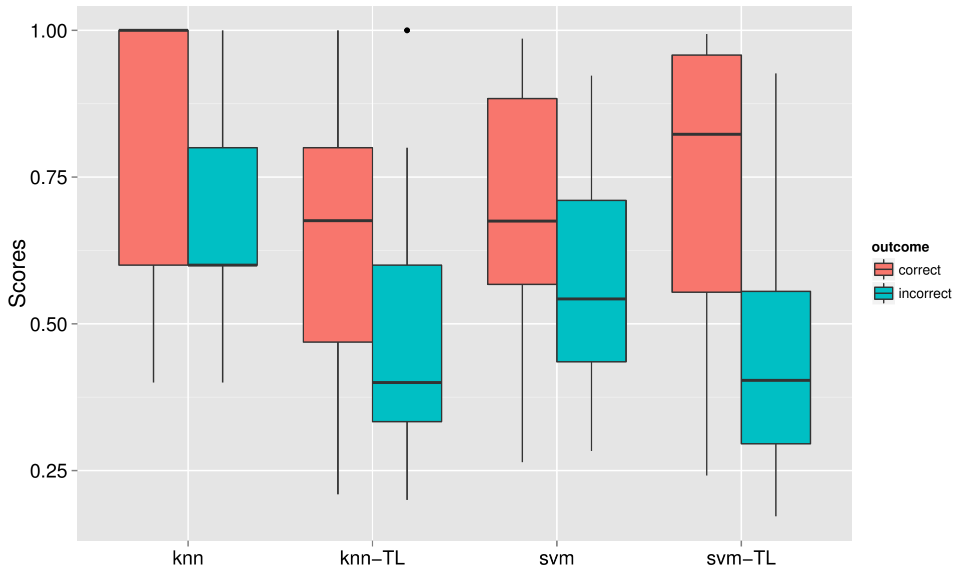

S4 File. Fig. A. Classification scores. Boxplots displaying the distribution of scores assigned to the unknown proteins in the mouse dataset for the k-NN, k-NN transfer learning (TL) algorithm, a Support Vector Machine (SVM) and the SVM TL classifiers. For each classifier the proteins have been split between those that have been classified as incorrect or correct according to known protein localisations found by a recent high resolution map of the mouse proteome [1].

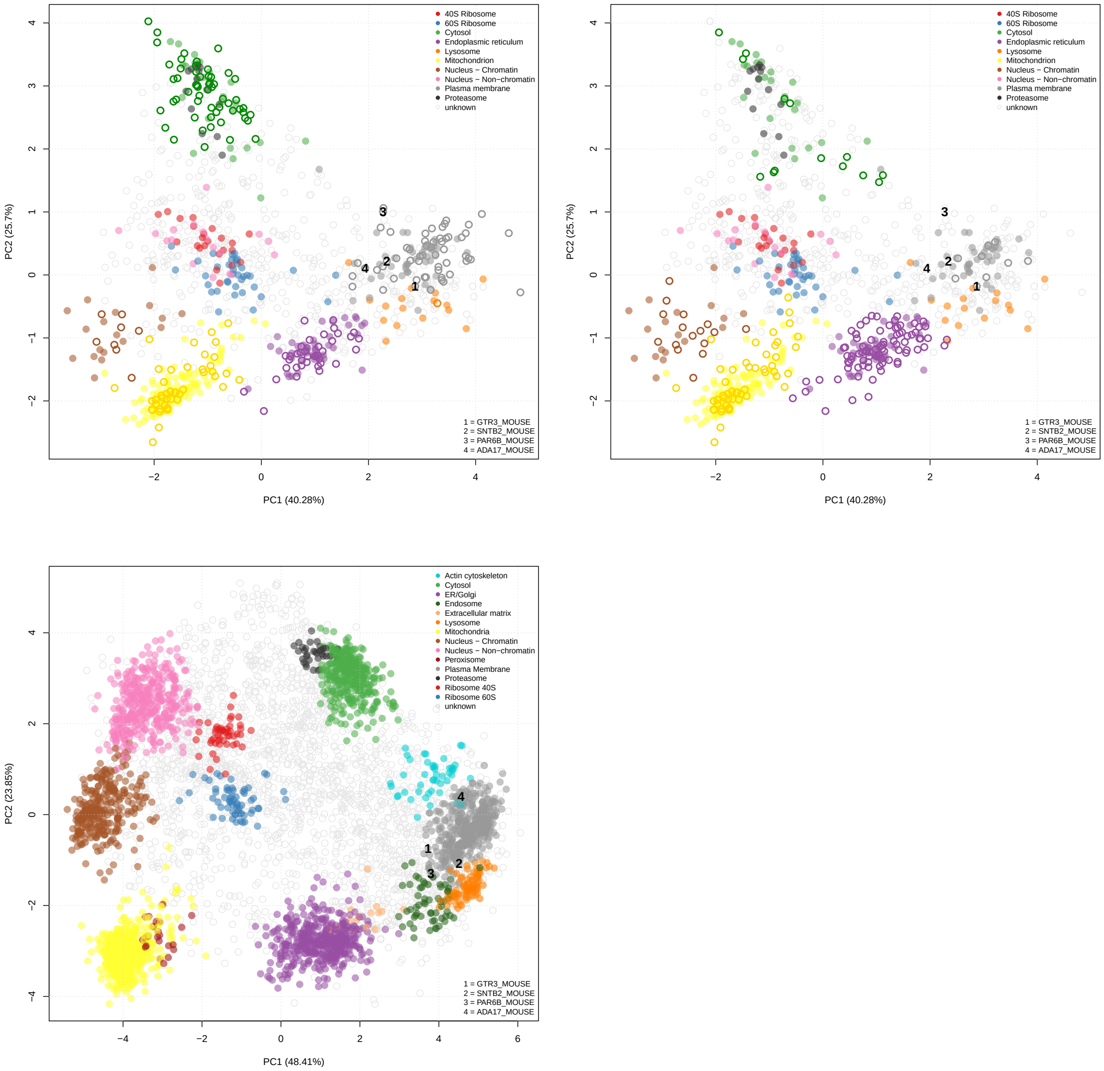

S4 File. Fig. B. Principal components analysis (PCA) plots of mouse stem cell datasets. The new localisations found using the SVM TL method (top left), the new localisations found using the k-NN TL method (top right) are highlighted on a plot of the original mouse stem cell dataset. The new sub-cellular map of the mouse stem proteome from the hyperLOPIT technology are displayed bottom left. Proteins are clustered according to the their density gradient distributions. Each point on the PCA plot represents one protein. Proteins are coloured according to sub-cellular location, circled proteins represent new assignments and filled circles represent markers used in classifier training. The 4 proteins GTR3_MOUSE, SNTB2_MOUSE, PAR6B_MOUSE and ADA17_MOUSE that were found in the SVM TL method and not in an SVM classification with LOPIT only are highlighted.

	RIB 40S	RIB 60S	CYT	ER	LYS	MT	CHR	NUC	PM	PROT	Total
SVM TL	0	0	56	37	1	45	8	0	57	0	204
SVM LOPIT only	0	4	0	46	0	47	9	5	38	0	149
Common assignments	0	0	0	28	0	42	7	0	31	0	108

S4 File. Table A. Sub-cellular protein assignments from using the SVM methods. The number of sub-cellular assignments of the unlabelled proteins amongst the 10 known sub-cellular classes that are common from application of a SVM (on LOPIT only) and the SVM transfer learning (TL) (using LOPIT and GO CC) for the mouse dataset, and also assignments that are only found in the SVM TL and only using a SVM with LOPIT alone. The classification thresholds for the SVM and SVM TL were 0.850 and 0.785 respectively, based on a FDR of 5%. RIB 40S = 40S ribosome, RIB 60S = 60S ribosome, CYT = Cytosol, ER = Endoplasmic reticulum, LYS = Lysosome, MT = Mitochondrion, CHR = Nucleus - Chromatin, NUC = Nucleus - Non-Chromatin, PM = Plasma membrane, PROT = Proteasome.

	RIB 40S	RIB 60S	CYT	ER	LYS	MT	CHR	NUC	PM	PROT	Total
k-NN TL	0	0	14	85	0	52	16	0	9	0	176
k-NN LOPIT only*	0	0	0	0	0	0	0	0	0	0	0
Common assignments*	0	0	0	0	0	0	0	0	70	0	0

S4 File. Table B. Sub-cellular protein assignments from using the *k*-NN methods. The number of sub-cellular assignments of the unlabelled proteins amongst the 10 known sub-cellular classes that are common from application of a *k*-NN (on LOPIT only) and the *k*-NN transfer learning (TL) (using LOPIT and GO CC) for the mouse dataset, and also assignments that are only found in the *k*-NN TL and only using a *k*-NN with LOPIT alone. The classification threshold for *k*-NN TL was 0.805, based on a FDR of 5%. *A FDR of 5% was not achievable for the *k*-NN with LOPIT only thus no classifications were made. The lowest FDR achievable for *k*-NN with LOPIT only was 15%, which resulted in a classification threshold score of 1 (i.e. all neighbours must share the same class label for a protein to be assigned a class). RIB 40S = 40S ribosome, RIB 60S = 60S ribosome, CYT = Cytosol, ER = Endoplasmic reticulum, LYS = Lysosome, MT = Mitochondrion, CHR = Nucleus - chromatin, NUC = Nucleus - Non-chromatin, PM = Plasma membrane, PROT = Proteasome.

		SVM TL										
		RIB 40S	RIB 60S	CYT	ER	LYS	MT	CHR	NUC	PM	PROT	unknown
	RIB 40S	0	0	0	0	0	0	0	0	0	0	0
	RIB 60S	0	0	0	0	0	0	0	0	0	0	0
	CYT	0	0	5	0	0	0	0	0	0	0	9
	ER	0	0	0	35	0	0	0	0	0	0	50
	LYS	0	0	0	0	0	0	0	0	0	0	0
k-NN TL	MT	0	0	0	0	0	44	0	0	0	0	8
	CHR	0	0	0	0	0	0	8	0	0	0	8
	NUC	0	0	0	0	0	0	0	0	0	0	0
	PM	0	0	0	0	0	0	0	0	9	0	0
	PROT	0	0	0	0	0	0	0	0	0	0	0
	Unknown	0	0	51	2	1	1	0	0	48	0	443

S4 File. Table C. Sub-cellular assignments of the unlabelled mouse proteins from the transfer learning (TL) methods. Contingency table showing the number of assignments of the unknowns in the mouse dataset using the k-NN TL and SVM TL methods among the sub cellular classes that were included in the training data. The classification score threshold for the k-NN was 0.805 and for the SVM TL method was 0.785 (based on a FDR of 5%), proteins that did not achieve greater than equal to these scores were set to unknown. Reassuringly we found no counts off the diagonal except those in the unknown columns and rows, again highlighting that the results between the two classifiers are in high agreement. We see that many more assignments are made using the SVM TL method, however the extra assignments gained using SVM TL are all found to be labelled as unknown in the k-NN TL method, and visa versa. RIB 40S = 40S ribosome, RIB 60S = 60S ribosome, CYT = Cytosol, ER = Endoplasmic reticulum, LYS = Lysosome, MT = Mitochondrion, CHR = Nucleus - chromatin, NUC = Nucleus - Non-Chromatin, PM = Plasma membrane, PROT = Proteasome.

References

[1] Christoforou A, Mulvey CM, Breckels LM, Hayward PC, Geladaki E, Hurrell T, et al. A draft map of the mouse pluripotent stem cell spatial proteome. Nat Commun; In Press.
