# Supplementary material for: Learning from Heterogeneous Data Sources: An Application in Spatial Proteomics
Source: PLoS Comput Biol. 2016 May 13;12(5):e1004920. doi: 10.1371/journal.pcbi.1004920 (PMC4866734; doi:10.1371/journal.pcbi.1004920)
Supplement: S5 File — A short comparison between k-NN transfer learning (TL) and SVM TL classifiers, including boxplots displaying the macro- and class-F1 scores for the k-NN TL and SVM TL experiments over the 100 test partitions on each dataset. This supplementary file also includes a comparison with Wu’s original k-NN transfer learning classifier and negative transfer effects are also described. (PDF) [file pcbi.1004920.s005.pdf]

S5 File: A comparison of transfer learning methods

k-NN TL versus SVM TL

We compared the macro- and class-F1 scores for the k-NN transfer learning (TL) and SVM TL methods on all datasets and found no single method outperformed the other, however, both methods outperformed using a classifier on one source alone. At the 0.01 significance level k-NN TL performed better overall than SVM TL for the callus dataset ($p = 4e^{-6}$) and SVM TL performs better than the k-NN TL method on the mouse dataset ($p = 6e^{-6}$). We also found that for the human, fly and roots datasets there was no significant difference in the performance between the two methods ($p = 0.07$, $p = 0.4$, $p = 0.01$, for each dataset respectively). Interestingly, the class-F1 scores showed that each TL method performed differently at the organelle level. For example, for the mouse dataset we found 4 of the 10 sub-cellular classes used in classifier creation performed significantly better with the k-NN TL, whereas another 4 of the sub-cellular classes performed better with SVM TL. For the 2 remaining classes both methods performed equally well. We see the same trend for all other datasets wherein no one method performs better on all sub-cellular classes than the other.

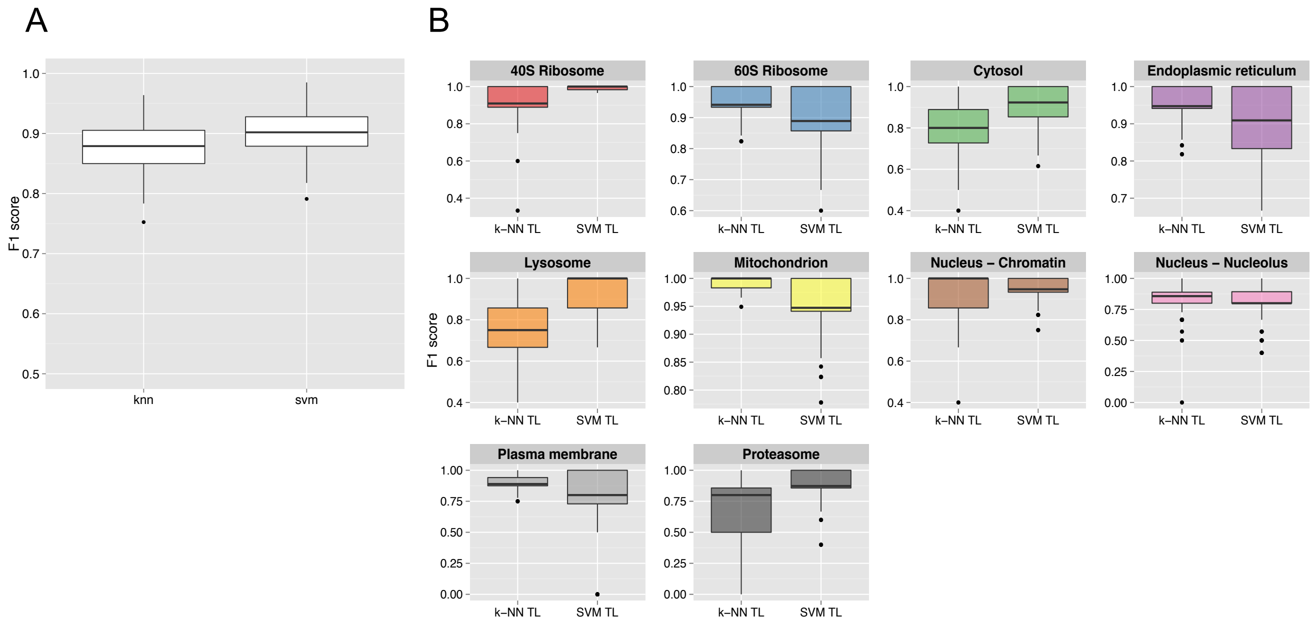

S5 File. Fig. A. Macro- and class-F1 results for the mouse dataset. Box plots displaying the macro-F1 (A) and class-F1 (B) scores for the *k*-NN transfer learning (TL) and SVM TL experiments over 100 test partitions on the mouse stem cell dataset.

	P value
40S ribosome	4e-12
60S ribosome	3e-07
Cytosol	3e-10
Endoplasmic reticulum	4e-05
Lysosome	3e-19
Mitochondrion	7e-10
Nucleus - Chromatin	1e-01
Nucleus - Non-chromatin	3e-01
Plasma membrane	1e-04
Proteasome	6e-08

S5 File. Table A. T-test results for the mouse dataset P values from an unpaired two-sample t-test (with unequal variance) used to determine if the populations means between the *k*-NN TL and SVM TL methods are significantly different from one another for each sub-cellular class in the mouse stem cell dataset.

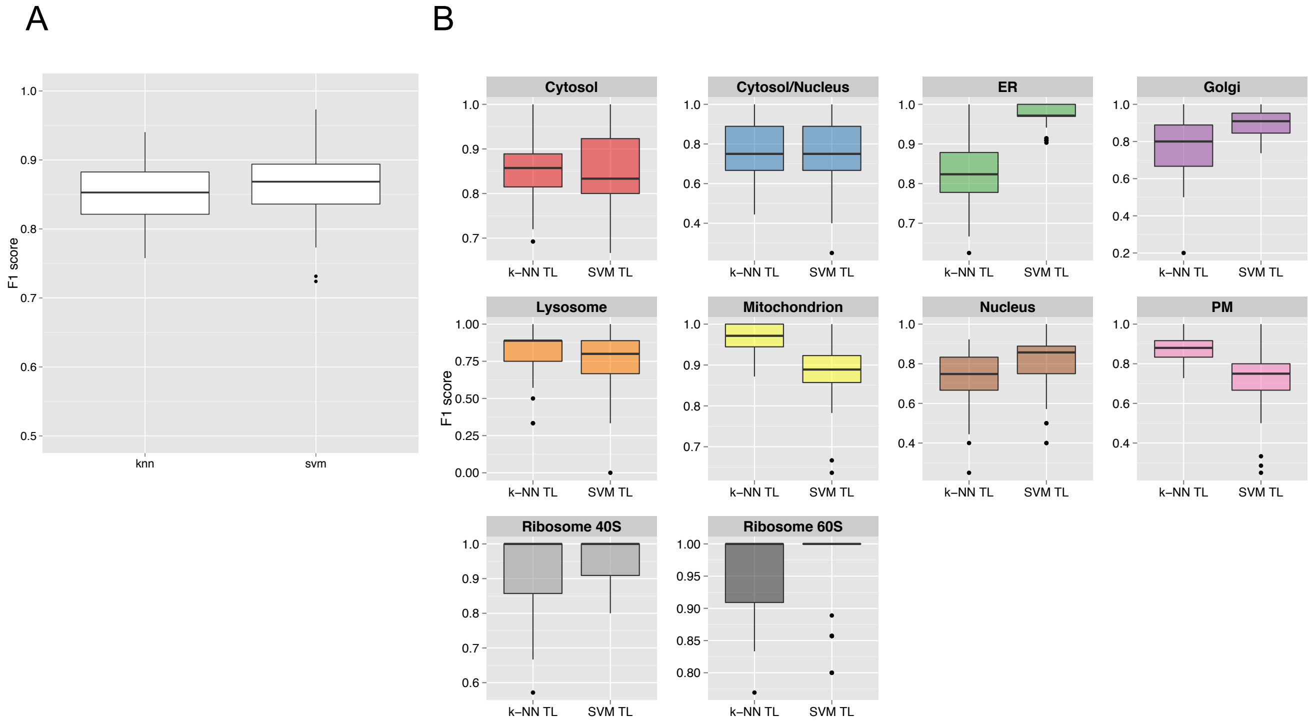

S5 File. Fig. B. Macro- and class-F1 results for the human dataset Box plots displaying the macro-F1 (A) and class-F1 (B) scores for the *k*-NN transfer learning (TL) and SVM TL experiments over 100 test partitions on the human LOPIT experiment

	p-value
Cytosol	7e-01
Cytosol/Nucleus	8e-01
ER	5e-32
Golgi	6e-13
Lysosome	3e-02
Mitochondrion	2e-21
Nucleus	2e-06
PM	6e-18
Ribosome 40S	5e-02
Ribosome 60S	9e-01

S5 File. Table B. T-test results for the human dataset *P* values from an unpaired two-sample t-test (with unequal variance) used to determine if the populations means between the *k*-NN TL and SVM TL methods are significantly different from one another for each sub-cellular class in the human dataset.

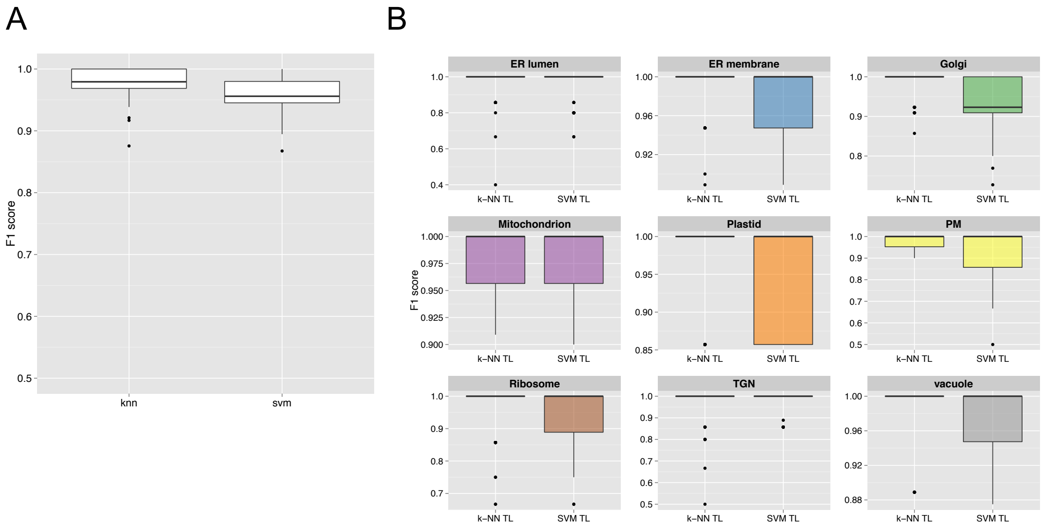

S5 File. Fig. C. Macro- and class-F1 results for the plant callus dataset. Box plots displaying the macro-F1 (A) and class-F1 (B) scores for the k-NN transfer learning (TL) and SVM TL experiments over 100 test partitions on the plant callus dataset.

	p-value
ER lumen	1e-01
ER membrane	3e-02
Golgi	4e-08
Mitochondrion	7e-02
Plastid	1e-02
PM	2e-05
Ribosome	5e-01
TGN	1e-01
vacuole	8e-02

S5 File. Table C. P values from an unpaired two-sample t-test (with unequal variance) used to determine if the populations means between the k-NN TL and SVM TL methods are significantly different from one another for each sub-cellular class in the plant callus dataset.

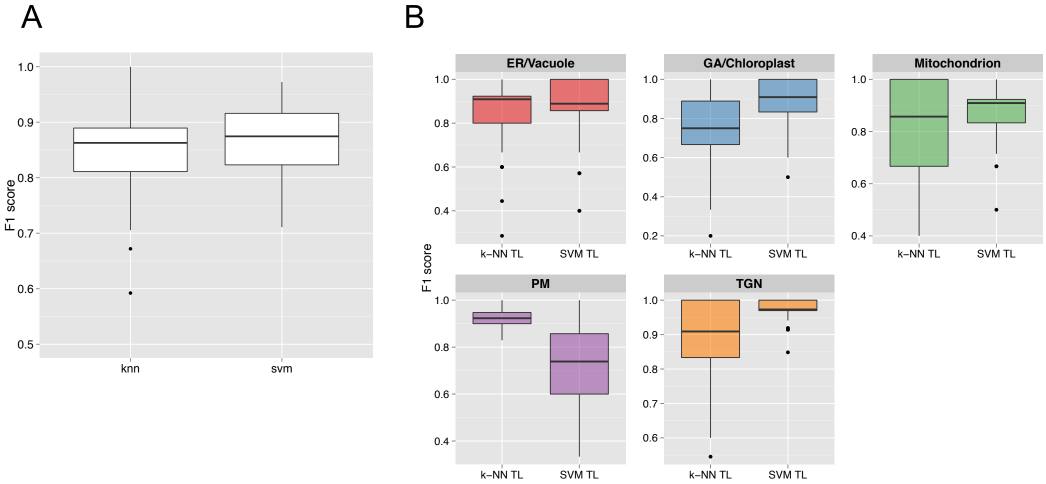

S5 File. Fig. D. Macro- and class-F1 results for the plants roots dataset. Box plots displaying the macro-F1 (A) and class-F1 (B) scores for the *k*-NN transfer learning (TL) and SVM TL experiments over 100 test partitions on the *Arabidopsis thaliana* roots dataset.

	p-value
ER/Vacuole	1e-01
GA/Chloroplast	1e-13
Mitochondrion	7e-04
PM	1e-22
TGN	6e-13

S5 File. Table D. T-test results for the plant roots dataset *P* values from an unpaired two-sample t-test (with unequal variance) used to determine if the populations means between the *k*-NN TL and SVM TL methods are significantly different from one another for each sub-cellular class in the plant roots dataset.

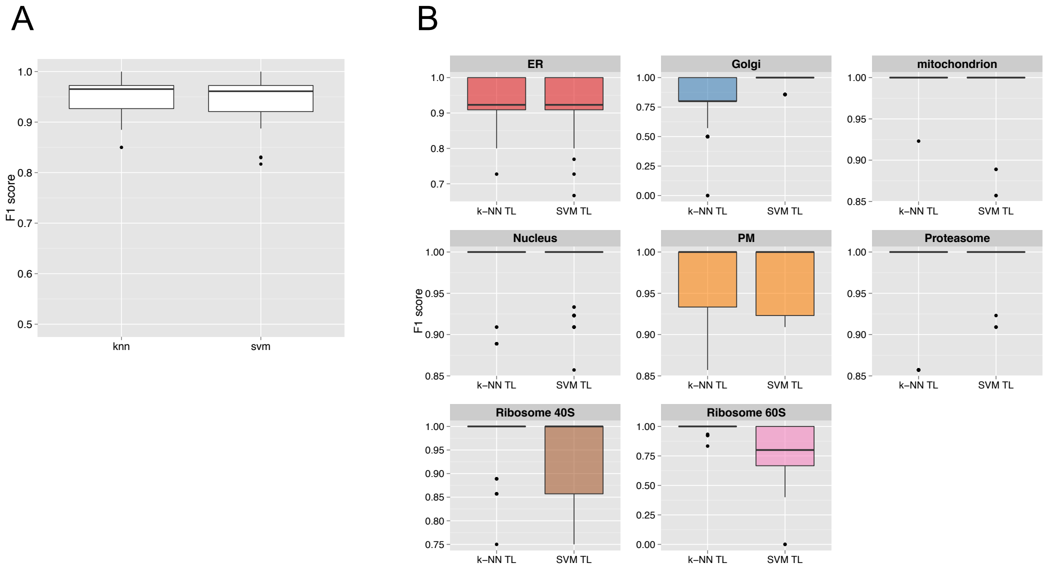

S5 File. Fig. E. Macro- and class-F1 results for the fly dataset. Box plots displaying the macro-F1 (A) and class-F1 (B) scores for the k-NN transfer learning (TL) and SVM TL experiments over 100 test partitions on the fly dataset.

	p-value
ER	8e-01
Golgi	8e-15
mitochondrion	1e-01
Nucleus	1e-01
PM	6e-01
Proteasome	6e-05
Ribosome 40S	2e-05
Ribosome 60S	9e-16

S5 File. Table E. T-test results for the fly dataset P values from an unpaired two-sample t-test (with unequal variance) used to determine if the populations means between the k-NN TL and SVM TL methods are significantly different from one another for each sub-cellular class in the fly dataset.

k-NN transfer learning: Wu's original method

In Wu and Dietterich's original application of transfer learning (TL) [1] the k-NN TL classifier only allowed weighting by data source and not on a data source **and** class-by-class basis. We have extended the usability of the method by incorporating a multi-class multi-data weighting schema to allow the integration of heterogeneous data types.

We compared Wu's k-NN TL method with own multi-class multi-data k-NN TL method (S5 File Figure F) (from herein we refer to these two methods as Wu and Breckels, for each TL method respectively). As described in the methods section of the manuscript to assess classifier performance we partitioned our labelled data into training and testing sets, and used the testing sets to assess the strength of our classifiers. Parameter optimisation was conducted on the labelled training data using 100 rounds of stratified 80/20 partitioning, in conjunction with 5-fold cross-validation in order to estimate the k-NN TL weights via a grid search. Comparing the macro-F1 scores at the 0.01 significance level we found that as per the Breckels method, Wu's method was better than using primary data alone for all datasets except the callus dataset (mouse $p = 2e^{-10}$, human $p = 9e^{-5}$, callus $p = 0.02$, roots $p = 9e^{-10}$, fly $p = 6e^{-6}$). We found that the Breckels k-NN TL classifier outperformed Wu's method for the mouse ($p = 4e^{-4}$) and roots dataset ($p = 4e^{-3}$). Both classifiers are implemented in the `pRoloc` package [2] in Bioconductor [3].

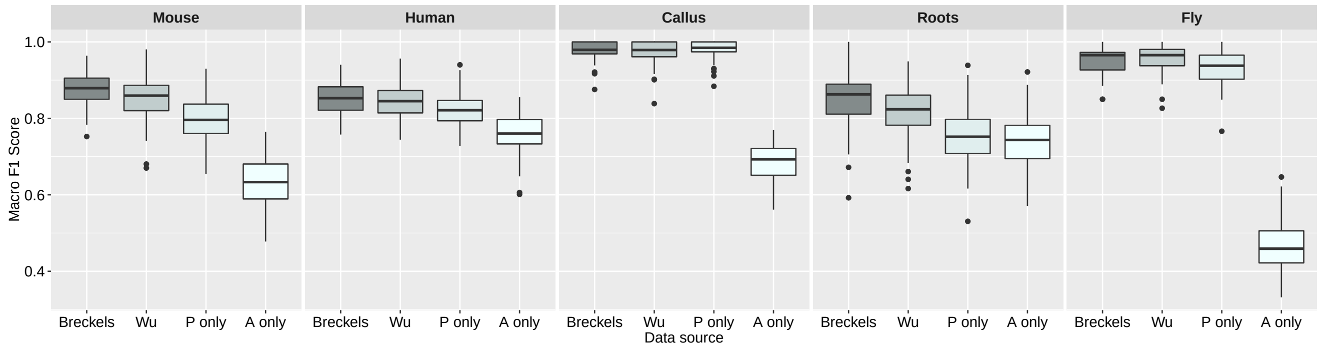

S5 File. Fig. F. A comparison of k-NN methods (Breckels, Wu and the baseline k-NN). Boxplots, displaying the estimated generalisation performance over 100 test partitions for the (i) Breckels' k-NN TL, (ii) Wu's k-NN TL, (iii) primary data only, and (iv) auxiliary data only, for each dataset.

Is is important to note that in two of the above cases, namely callus and fly, learning from auxiliary data has either limited (fly), or no effect at all (callus) because the resolution in the primary data is already excellent (the primary F1 scores are close to 1). Neither Wu nor Breckels algorithms can bring much using TL for these cases, and hence the comparison of Wu's and Breckel's k-NN are not particularly telling here. For the case where improvement is possible (fly), both algorithms result in an increase in performance, but scope for improvement is so limited that it is impossible to separate them. If we consider the other datasets (mouse, human and roots), where integration of primary and auxiliary data is most useful, our k-NN TL algorithm outperforms Wu's original algorithm in 2 out of 3 cases (mouse and roots).

In addition, we found that our SVM TL algorithm outperforms Wu's k-NN algorithm (S5 File Fig. G) in all 3 cases: mouse ($p = 7e^{-13}$), roots ($p = 7e^{-8}$) and human ($p = 4e^{-3}$).

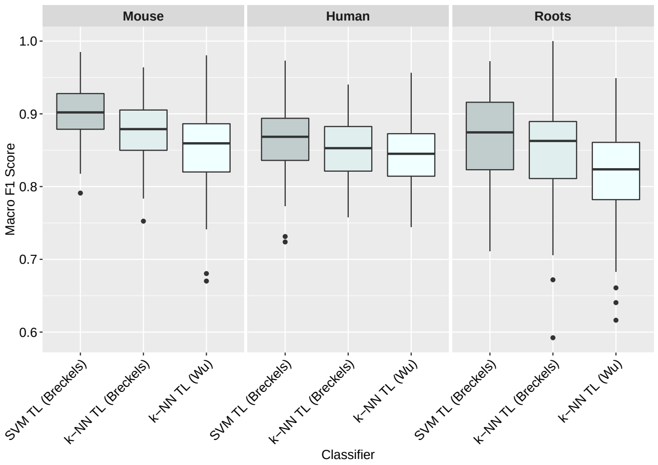

S5 File. Fig. G. A comparison of all TL classifiers (SVM TL and the *k*-NN TL classifiers.) Boxplots, displaying the estimated generalisation performance over 100 test partitions for (i) Breckels' SVM TL, (ii) Breckels' *k*-NN TL, (ii) Wu's *k*-NN TL, for each dataset.

Negative transfer

Negative transfer is a paradigm in machine learning used to describe the situation when (often irrelevant) information is transferred from an auxiliary source which results in a decrease in the performance of the learner. A major hurdle that one faces in developing successful transfer learning methods is how to minimise the negative transfer paradigm [4]. In a review of transfer learning [5] Pan and Yang provide an introduction to transfer learning in which they address three key issues (1) what to transfer, (2) how to transfer, (3) when to transfer, and how these relate to negative transfer and similarity between source/target domains and tasks. Olivas et al [4] also provide insight on to how to avoid negative transfer and choose source tasks wisely. One such way is to manually select what to transfer, which is possible with the two TL methods presented here by manually setting the class-weights in the *k*-NN TL classifier and data-specific SVM parameters in the SVM TL classifier.

We observe some negative transfer events on a class-specific basis. For example, from examining the class-F1 scores for the mouse dataset we see from Fig. 2 (bottom) in the main body of the manuscript, *k*-NN TL does not perform as well for the lysosome to using primary alone. We find however that a t-test shows that this difference is not significant at 0.01 ($p = 0.07$). We observe the converse for the proteasome, in terms of auxiliary performance, wherein adding primary information decreases the performance of the auxiliary data alone ($p = 6e^{-3}$, for combined versus auxiliary). As mentioned above, one of the advantages of the k-NN TL algorithm is the ability to set the weights for these organelles manually, so we can limit the cases where negative transfer may happen.

We have found in previous tests that straightforward concatenation of the primary and auxiliary data i.e. where no data is weighted, for many cases fails and indeed we see strong negative-transfer effects. Following our usual protocol for testing classifier performance (as detailed in the methods), the resultant 100 macro-F1 scores from straightforward concatenation was compared to those obtained from training on primary alone and to training on auxiliary alone (S5 File Figure H). We find that simple concatenation of the primary and auxiliary data results in a significant decrease in classifier performance for some datasets compared to using just primary data alone, as seen in, for example, the human ($p = 2e^{-20}$) and callus ($p = 5e^{-50}$) datasets.

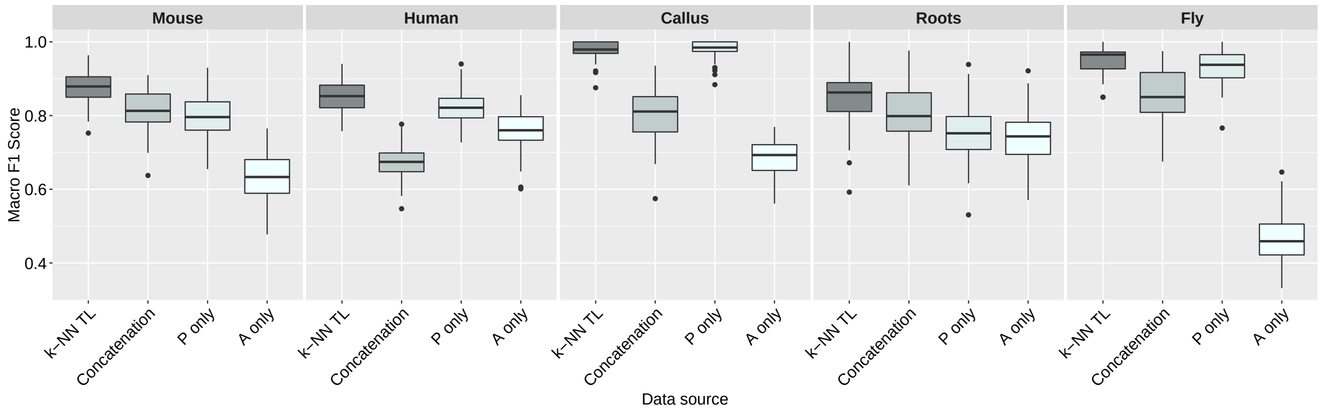

S5 File. Fig. H. Negative transfer effects from straightforward data combination. Boxplots displaying the macro F1 scores over 100 test partitions for the (i) k-NN classifier, (ii) straightforward concatenation of primary and auxiliary data (GO) sources, (iii) primary data only, and (iv) auxiliary data only, for each dataset.

References

[1] Wu P, Dietterich TG. Improving SVM Accuracy by Training on Auxiliary Data Sources. In: Proceedings of the 21st International Conference on Machine Learning (ICML); 2004.

[2] Gatto L, Breckels LM, Wieczorek S, Burger M, Lilley KS. Mass-spectrometry based spatial proteomics data analysis using pRoloc and pRolocdata. Bioinformatics. 2104;30(9):1322–1324.

[3] Gentleman RC, Carey VJ, Bates DM, Bolstad B, Dettling M, Dudoit S, et al. Bioconductor: open software development for computational biology and bioinformatics. Genome Biol. 2004;5(10):–80.

[4] Olivas ES, Guerrero JDM, Martinez-Sober M, Magdalena-Benedito JR and Serrano Lopez AJ. Handbook of Research on Machine Learning Applications and Trends: Algorithms, Methods, and Techniques (2 Volumes) 1st Edition. IGI Global, 2010.

[5] Pan SJ and Yang Q. A Survey on Transfer Learning IEEE Trans. on Knowl. and Data Eng. 2010;22(10) 1345–1359
