# Supplementary material for: Learning from Heterogeneous Data Sources: An Application in Spatial Proteomics
Source: PLoS Comput Biol. 2016 May 13;12(5):e1004920. doi: 10.1371/journal.pcbi.1004920 (PMC4866734; doi:10.1371/journal.pcbi.1004920)
Supplement: S6 File — Tables displaying the dimensions for each primary and auxiliary dataset, including the total number of proteins identified in each LOPIT dataset and number of known markers of sub-cellular protein location. (PDF) [file pcbi.1004920.s006.pdf]

S6 File: Summary Statistics

This supplement contains summary statistics for all datasets used in the manuscript. The full datasets are freely distributed as part of the Bioconductor [1] `pRolocdata` data package [2].

Labelled instances (markers)										Unlabelled	Total
RIB 40S	RIB 60S	CYT	ER	LYS	MT	CHR	NUC	PM	PROT		
23	36	25	44	16	150	18	18	43	14	722	1109

S6 File. Table A. Proteins identified in the mouse stem cell dataset including markers of protein sub-cellular localisation. RIB 40S = 40S ribosome, RIB 60S = 60S ribosome, CYT = Cytosol, ER = Endoplasmic reticulum, LYS = Lysosome, MT = Mitochondrion, CHR = Nucleus - Chromatin, NUC = Nucleus - Non-chromatin, PM = Plasma membrane, PROT = Proteasome.

Labelled instances (markers)												Unlabelled	Total
CHR	CYT	CYT/NUC	END	ER	GA	LYS	MT	NUC	PM	RIB 40S	RIB 60S		
11	60	22	12	36	24	22	89	27	54	18	29	967	1371

S6 File. Table B. Proteins identified in the human dataset including markers of protein sub-cellular localisation. CHR = Chromatin associated, CYT = Cytosol, CYT/NUC = Cytosol or nucleus localised, END = Endosome, ER = Endoplasmic reticulum, GA = Golgi apparatus, LYS = Lysosome, MT = Mitochondria, NUC = Nucelus, PM = Plasma membrane, RIB 40S = Ribosome 40S, RIB 60S = Ribosome 60S.

Labelled instances (markers)									Unlabelled	Total
ER L	ER M	GA	MT	PL	PM	RIB	TGN	VA		
14	45	28	55	20	46	19	13	21	428	689

S6 File. Table C. Proteins identified in the plant callus dataset including markers of protein sub-cellular localisation. ER L = Endoplasmic reticulum lumen, ER M = Endoplasmic reticulum membrane, GA = Golgi apparatus, MT = Mitochondria, PL = Plastid, PM = Plasma membrane, RIB = Ribosome, TGN = *Trans*-Golgi network, VA = Vacuole

Labelled instances (markers)					Unlabelled	Total
ER/VA	GA/CHL	MT	PM	TGN		
26	21	20	89	29	1155	1340

S6 File. Table D. Proteins identified in the plant roots dataset including markers of protein sub-cellular localisation. ER/VA = Endoplasmic reticulum or vacuole, GA/CHL = Golgi apparatus or chloroplast, MT = Mitochondria, PM = Plasma membrane, TGN = *Trans*-Golgi network, VA = Vacuole

Labelled instances (markers)											Unlabelled	Total
CTK	ER	GA	LYS	MT	NUC	PER	PM	PROT	RIB 40S	RIB 60S		
7	28	13	8	29	21	4	34	15	20	32	677	888

S6 File. Table E. Proteins identified in the fly dataset including markers of protein sub-cellular localisation. CTK = Cytoskeleton, ER = Endoplasmic reticulum, GA = Golgi apparatus, LYS = Lysosome, MT = Mitochondria, NUC = Nucleus, PER = Peroxisome, PM = Plasma membrane, PROT = Proteasome, RIB 40S = Ribosome 40S, RIB 60S = Ribosome 60S

Dataset	# proteins		# features			
	Labelled	Unlabelled	Primary: LOPIT	Auxiliary: GO CC	HPA	YLoc
Mouse	387	722	8	314		387
Human*	404	967	8	355	18	
Fly	211	677	4	138		
Plant callus	261	428	16	70		
Plant roots	185	1155	6	153		

S6 File. Table F. Data dimensions for proteins identified the primary and auxiliary datasets. LOPIT: Localisation of organelle proteins using isotope tagging, GO CC: Gene ontology cellular compartment, HPA: Human Protein Atlas, YLoc: YLoc sequence and annotation features). *Only information from the HPA for 191 of the labelled markers was available, and for 479 of the unlabelled proteins.

References

[1] Gentleman RC, Carey VJ, Bates DM, Bolstad B, Dettling M, Dudoit S, et al. Bioconductor: open software development for computational biology and bioinformatics. Genome Biol. 2004;5(10):–80. Available from: `http://dx.doi.org/10.1186/gb-2004-5-10-r80`.

[2] Gatto L, Breckels LM, Wieczorek S, Burger M, Lilley KS. Mass-spectrometry based spatial proteomics data analysis using pRoloc and pRolocdata. Bioinformatics. 2104;30(9):1322–1324. Available from:`http://www.bioconductor.org/packages/release/data/experiment/html/pRolocdata.html`
